# Navigated Transtubular Extraforaminal Decompression of the L5 Nerve Root at the Lumbosacral Junction: Clinical Data, Radiographic Features, and Outcome Analysis

**DOI:** 10.1155/2016/3487437

**Published:** 2016-04-04

**Authors:** P. Stavrinou, R. Härtl, B. Krischek, C. Kabbasch, A. Mpotsaris, R. Goldbrunner

**Affiliations:** ^1^Department of Neurosurgery, University Hospital of Cologne, 50937 Cologne, Germany; ^2^Department of Neurological Surgery, Weill Cornell Medical College, New York, NY 10021, USA; ^3^Department of Radiology and Neuroradiology, University Hospital of Cologne, 50937 Cologne, Germany

## Abstract

*Purpose*. Extraforaminal decompression of the L5 nerve root remains a challenge due to anatomic constraints, severe level-degeneration, and variable anatomy. The purpose of this study is to introduce the use of navigation for transmuscular transtubular decompression at the L5/S1 level and report on radiological features and clinical outcome.* Methods*. Ten patients who underwent a navigation-assisted extraforaminal decompression of the L5 nerve root were retrospectively analyzed.* Results*. Six patients had an extraforaminal herniated disc and four had a foraminal stenosis. The distance between the L5 transverse process and the para-articular notch of the sacrum was 12.1 mm in patients with a herniated disc and 8.1 mm in those with a foraminal stenosis. One patient had an early recurrence and another developed dysesthesia that resolved after 3 months. There was a significant improvement from preoperative to postoperative NRS with the results being sustainable at follow-up. ODI was also significantly improved after surgery. According to the Macnab grading scale, excellent or good outcomes were obtained in 8 patients and fair ones in 2.* Conclusions*. The navigated transmuscular transtubular approach to the lumbosacral junction allows for optimal placement of the retractor and excellent orientation particularly for foraminal stenosis or in cases of complex anatomy.

## 1. Introduction

Compression of the L5 nerve root at the lumbosacral junction is a rare occurrence and is usually due to extraforaminal lumbar disc herniation (ELDH) or (extra)foraminal stenosis (EFS). ELDH or far-lateral disc herniations are relatively rare, accounting only for 1–12% of all lumbar disc herniations [1]. Extraforaminal disc herniations at the L5/S1 level are the most uncommon type, with reported rates from the magnetic resonance imaging era varying between 6.5 and 25% of all ELDH, while the (extra)foraminal stenosis is an underreported pathology and it is only recently that it has been studied as a distinct causative factor [2]. Although many surgical techniques have been described for the treatment of ELDH and EFS, there is rather a consensus that the extraforaminal, muscle-splitting, minimally invasive techniques come with many advantages for the stability of the lumbar spine and the postoperative course.

The short distance between the broad L5 transverse process and the sacral ala, the broader pars interarticularis of the L5 lamina, the coronally oriented facet joints, and the iliac crest laterally make the operative corridor very narrow, particularly in older patients with collapsing of the L5/S1 disc and facet hypertrophy. Adding to the anatomic constrains, the fact that the ELDH and EFS at the lumbosacral level are rare occurrences limits the surgeons' exposure and the possibility to become familiar with the extraforaminal decompression of the L5 spinal nerve. In this report, we introduce and discuss the combination of the neuronavigation with a minimally invasive intermuscular approach utilizing a tubular retractor and the operating microscope.

## 2. Materials and Methods

Ten consecutive patients who underwent a navigation-assisted transmuscular transtubular approach for extraforaminal compression of the L5 nerve root were retrospectively analyzed. The records were reviewed for demographic data; type of L5 compression (herniated disc or extraforaminal stenosis); pre- and postoperative clinical symptoms, as well as duration of symptoms and type of conservative treatment used; intraoperative data; length of stay; and duration of follow-up. Pain evaluation and neurological assessment were conducted preoperatively as well as immediately postoperatively and at the time of the last follow-up examination, using the numeric rating scale (NRS), the validated German version of the Oswestry Disability Index (ODI-D), and the Macnab scale [3, 4]. All patients underwent a preoperative spiral CT and MRI imaging of the lumbar spine. The type of L5 root compression was classified as either “disc herniation”—when there was an extraforaminal rupture or a contained disc herniation—or as “(extra)foraminal stenosis” for the cases where there was a diffuse disc bulging with variable grade of segmental degeneration, causing intra- and/or extraforaminal compression of the nerve. Using reconstructed images on axial, sagittal, and coronal levels, two independent raters (P. Stavrinou and C. Kabbasch) measured the optimal angle of approach at an axial level; this was defined as the angle through which Kambin's triangle (i.e., the triangular space over the dorsolateral disc, the exiting nerve root, and the dura) could be approached with minimum interference from the iliac crest and the lateral facet. The distance between the L5 transverse process and the para-articular notch of the sacrum was also measured.

**Figure 1:**
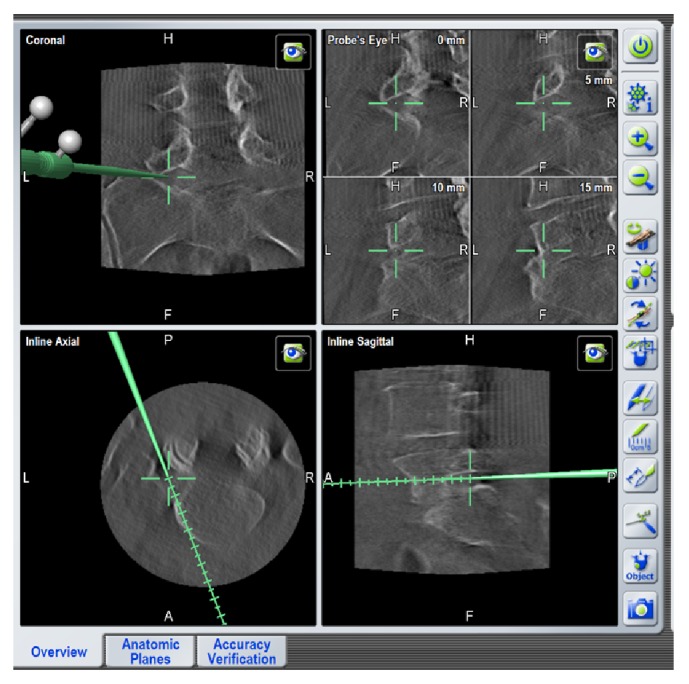
Screenshot from the Brainlab neuronavigation system. Orientation and identification of the anatomic landmarks with the use of pointer through the tubular retractor.

**Figure 2:**
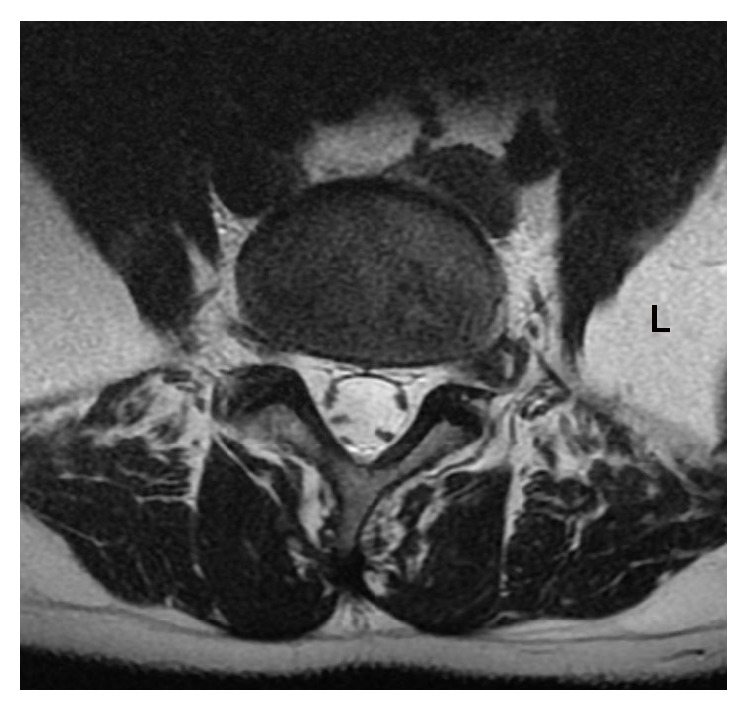
Axial T2-weighted MRI reveals extraforaminal ruptured disc on the left side (patient number 5). Therapy consists of removal of the fragmented disc segment without bone removal or discectomy. Navigation allows for a safe transmuscular approach.

After induction of general anesthesia, patients were positioned prone on the carbon operating table with chest and pelvic bolsters. In lateral view, a 3 mm spinal process screw (25 mm length) was inserted in percutaneous fashion in the spinal process of L4 for fixation of the reference array. Using a Ziehm Vario 3D C-Arm (Ziehm Imaging GmbH, Germany), a 3D scan of the patient was acquired and the data set was transferred automatically to the navigation system. Using the Brainlab pointer and a virtual tip offset of variable length, the entry point and surgical trajectory were identified. A paramedian incision was made through the skin and fascia and, with the pointer as a guide, the initial dilator was passed through the paraspinal muscles until bone contact was made. After sequential dilation and insertion of the 19 mm dilator, the DePuy Insight Retractor was inserted and connected to the flex arm. At this point the position of the retractor was controlled with the navigation pointer and, if necessary, adjustments were made (Figure 1). Even before dissection of the soft tissue, the anatomic landmarks were identified with the pointer. The remnant soft tissue was removed under microscope until visualization of the lateral superior articular process of S1. Depending on the anatomical constrains in the individual patient, a drill was used to shave down the lateral facet and, if needed, the inferior margin of the L5 transverse process. Cranial angulation of the retractor as planned preoperatively helped to avoid the sacral ala in all cases. Further surgical strategy was adapted to the underlying pathology; in cases of a ruptured disc the nerve root was identified and retracted using a dissector, taking care not to injure the radicular vessels (Figure 2). The disc fragment was mobilized and removed with a hook followed by a conservative discectomy, meaning removal of disc material that may not be herniated yet but is in continuity with the herniated fragment or hanging loose in the intervertebral space. Removal of the whole disc material was not attempted. In cases of foraminal stenosis, the bony decompression is more important, and the drilling of the bony confines was more generous. After identification of the exiting nerve root, the osteophytes were also removed. Due to annular bulging, a discectomy was performed along the intra- and extraforaminal portion of the disc. At the end, the exploration along the nerve route up to the lumbosacral tunnel showed no signs of nerve impingement. Hemostasis was performed mainly with copious irrigation and bipolar cauterization as the retractor is slowly removed. No drainage was used and the wound was closed in standard fashion. Patients were mobilized on the first postoperative day.

## 3. Results

Of the ten patients treated, there were seven men and three women. The mean age was 50.2 (±12.1) years (range 35–75 years). All patients complained about unilateral radicular pain in the distribution of the fifth lumbar nerve root. The right side was affected in three (27.3%) and the left side in seven (63.6%) cases. All patients were initially treated conservatively with various methods, usually prescribed by the family physician. The duration of symptoms was highly variable, with seven patients complaining of acute onset and duration of pain of about four weeks and three patients reporting an aggravation of a familiar chronic radicular pain (Table 1). The pain was reported as being severe in all cases (mean NRS 8.6). Five of the patients complained about hypoesthesia or paresthesia along the L5 dermatome, while three of them also experienced a foot or toe flexion paresis. The mean preoperative ODI score was 64 (±19.4, range 30–94). Seven out of ten patients were overweight and the mean body mass index (BMI) was 27.7 (range 23.4–38).

Based on preoperative imaging as well as intraoperative findings, six patients had a disc herniation and four had a foraminal stenosis. On CT, the mean distance between the two adjacent transverse processes at the L4/5 level was twice the one measured between the L5 transverse process and the para-articular notch of the sacrum at the L5/S1 level, indicating a twice as narrow “working canal” through which the L5 nerve root must be decompressed. (*M*_L4/5_ = 21.6 versus *M*_L5/S1_ = 10.5 mm).  Table 2 summarizes relevant measurement regarding the working canal and the optimal angle of approach.

**Figure 3:**
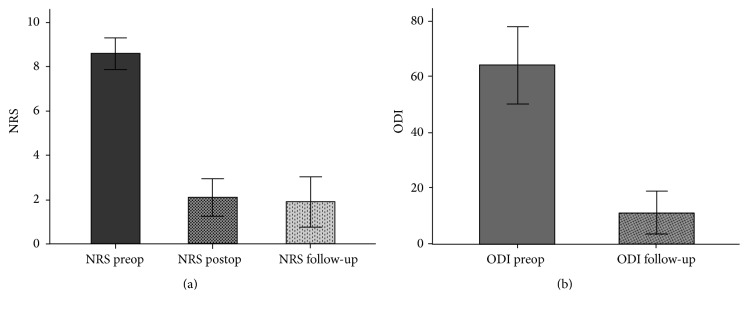
(a) Mean NRS preoperatively, postoperatively, and on follow-up. (b) Mean ODI-D preoperatively and on follow-up. Error bars show 95% CI.

Mean operation time was 130.5 minutes (range 98–217 minutes) with no significant difference between herniated disc and foraminal stenosis cases (*M*_dh_ = 137.8 versus *M*_fs_ = 119.5 min), and mean blood loss was 77 mL (range 50–150 mL). In only one patient with a disc protrusion was drilling of the lateral facet necessary; however, all foraminal stenosis cases required drilling of the facet joint and the caudal surface of the L5 transverse process. Conservative discectomy was performed in all cases. There were no intraoperative complications. One patient (patient number 4, Table 1) developed an early disc herniation recurrence which required a revision surgery, and another had dysesthesia along the L5 dermatome that resolved after three months. Mean hospital stay was five days. All patients reported significant relief of their preoperative pain. NRS at discharge averaged at 2.1. The patients were followed up for a mean of 22 (8–38) months. All patients that had a preoperative motor deficit improved at least one grade on the muscle strength scale, while the sensory deficits resolved in all cases but one, in which a mild hypoesthesia persisted (Table 1). NRS on follow-up remained low (*M* = 1.9). Pain postoperatively, both directly after the operation and on follow-up, was significantly improved compared to preoperatively (*F*_(2,18)_ = 115, *p* < 0.001) (Figure 3(a)). There was also a significant improvement of the ODI score at the final follow-up compared to preoperative scoring (*M* = 11, *F*_(1.9)_ = 142.4, *p* < 0.001) (Figure 3(b)). General clinical outcome based on the Macnab scale was excellent in three (30%) patients, good in five (50%), and fair in two (20%). Nine out of ten patients returned to their jobs or resumed their preoperative activities.

## 4. Discussion

The extraforaminal disc herniation and (extra)foraminal stenosis at the lumbosacral junction are diagnostic and therapeutic challenges. The rarity of the ELDH and even more that of the EFS make these pathologies easy to overlook, often resulting in failed back syndrome [5]. Conservative treatment remains the first-line therapy, but it usually has limited effect on the marked pain due to the involvement of the dorsal root ganglion [6]. In our series, six out of ten patients had at least three CT-guided periradicular injections of the L5 root without relevant improvement (Table 1). For patients that fail conservative treatment, various surgical techniques have been discussed. Midline approaches with muscle retraction and partial facetectomy are widely employed, and the advantages and disadvantages have frequently been discussed [6–9]. In our opinion, the biggest advantage of the midline approach is familiarity with the procedure. The problem is that the anatomic features of the lumbosacral junction make this advantage less relevant. The coronally oriented facet joints; the wider pedicles; the shorter length from the caudal transverse process to the superior edge of the inferior articular process; and, more importantly, the prominent iliac crest make the necessary extended muscle mobilization and subperiosteal dissection extremely arduous. These difficulties become more evident in cases with foraminal stenosis. The foraminal stenosis in these patients is usually the combined result of the degenerative changes of the lumbosacral junction, that is, facet hypertrophy, disc collapse, osteophytes, and so forth. Decompression of the intra- and extraforaminal space in these cases is only possible with significant removal of the facet joint, which in turn can cause further accelerated degeneration with secondary instability and chronic lumbar pain [9]. On the other hand, since both the view and the approach are perpendicular, cautious reduction of the facet joint—in an attempt to prevent instability—can lead to remaining foraminal stenosis, particularly in cases with significant segmental degeneration or intraforaminal disc bulging.

The transmuscular paramedian approach to the extraforaminal space, first described by Wiltse and Spencer, requires no muscle detachment and less bone removal [10, 11]. With time, even more refined, minimally invasive techniques were developed, but, initially, the L5/S1 segment was still considered more or less* terra nullius*: Foley et al. described their experience with 11 patients with far-lateral disc herniations but did not operate at the lumbosacral junction [12]. Cervellini et al. presented their series of 17 patients but did not operate on the lumbosacral junction either, claiming that application of this technique is not possible at the L5-S1 level due to anatomical constrains [13]. Grainer-Perth et al. reported on 15 patients with ELDH that were treated with microendoscopic technique, but only one of them was operated on at the L5/S1 level with moderate results [14]. After 2007, several studies reported their results with minimally invasive extraforaminal decompression of the L5 nerve: Kotil et al. analyzed 14 patients and demonstrated effective decompression of the L5 root in 13 of them [15]. Pirris et al. presented their results on four patients using a muscle-splitting technique and a microscope, while Zhou et al. utilized a METRx intertransverse decompression on five patients with excellent outcomes in three and good outcomes in two [16, 17]. D. Y. Lee and S.-H. Lee presented a large series with 65 patients who underwent microscopic decompression at the L5-S1 level [18]. They reported disappointing results—significantly worse than those presented in most studies—and attributed those to an incorrect understanding of the anatomy at the lumbosacral junction, especially in cases with foraminal compression of the L5 nerve.

**Figure 4:**
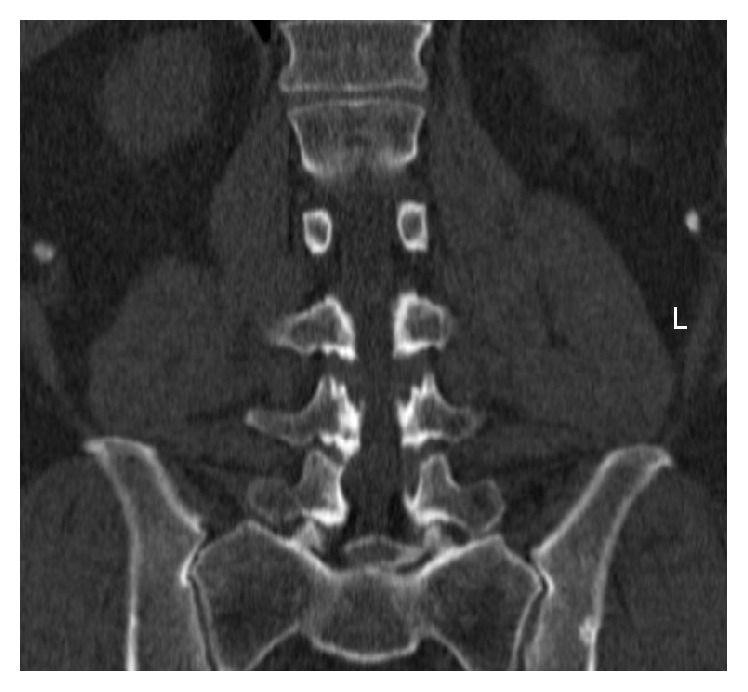
Extraforaminal stenosis at the lumbosacral junction. The degeneration of the lower lumbar spine leads to collapsing of the L5-S1 segment, contact between the sacrum and the L5-transverse process, and a very narrow operating window on the symptomatic side (left) that requires significant bone removal (patient number 3).

The literature suggests very good clinical results with extraforaminal approaches—both microscopic and endoscopic—as long as there is adequate decompression of the affected nerve. The challenge is achieving an adequate decompression while at the same time avoiding unnecessary bone and soft tissue trauma all through a very narrow and deep corridor and a highly variable anatomy. The endoscope is certainly a valuable tool in experienced hands, but the learning curve is extensive. While most surgeons are familiar with the use of the microscope, operating through a tubular retractor limits the surgical exposure and deprives the surgeon of the familiar landmarks. Additionally, the rarity of the extraforaminal disc herniation and stenosis of the L5-S1 level makes it rather hard to accumulate experience, particularly in low-volume departments. The use of navigation helps circumvent that problem. It allows an optimal placement of the tubular retractor, even in the absence of any visual anatomical landmarks and an estimation of the distance to the structures of interest. Another significant advantage is the assessment of necessary bone removal in cases of foraminal stenosis or challenging anatomy: the inability to decompress the intraforaminal area has been considered one of the limitations of the approach (Figure 4). Navigation allows for a precise assessment of the necessary degree of facet resection, thus minimizing the risk for residual stenosis or instability.

Our results are comparable to those in the literature. It should be noted that most studies refer only to extraforaminal disc herniation. Very few previous studies have included patients with (extra)foraminal stenosis, sometimes with unfavorable results [18, 19]. In our small series, good results were achieved despite the fact that four out of ten patients had extra/foraminal stenosis. Moreover, none of the patients developed low back pain or did eventually require fusion. We believe this is also due to precise reduction of the surrounding bony structures, tailored to each patient's individual pathology and anatomy with the aid of the navigation. The mean blood loss of 77 mL is comparable with that described in the literature [16–18, 20], but our mean surgical time of 130.5 minutes is rather long. This could be attributed to three factors: first, the fact that four of our patients had an EFS which is more complex to treat than a disc herniation; second, most of our patients being overweight; and finally, the implementation of the navigation itself.

## 5. Conclusions

The transmuscular extraforaminal decompression of the L5 nerve root at the lumbosacral junction is an effective and minimally invasive technique. The aid of the navigation allows for a patient-tailored approach and adequate surgical exploration even in face of complex lesion anatomy.

## Figures and Tables

**Table 1:** Case summaries. LDH indicates lumbar disc herniation; RP: radicular pain; FF: foot flexion paresis; BTF: big-toe flexion paresis; SD: sensory deficit; PA: per os analgetics; IV: intravenous analgetics; Ph: physiotherapy; PRI: periradicular infiltration.

Case number	Age (yrs), sex	Pathology	Preoperative symptoms	Duration of symptoms (weeks)	VAS (preop)	Conservative treatment	VAS (postop-follow-up)	ODI	Macnab	Complications
1	42, F	LDH	RP	5	7	PA, IV	3-2	6	2	None
2	58, M	LDH	RP, FF(4), SD	48	10	PA, IV, PRI	2-5	32	3	Dysesthesia (3 months)
3	62, F	Stenosis	RP, FF(3), BTF(4)	4	8	PA, Ph	1-0	2	2	None
4	35, F	LDH	RP	2	9	PA, IV	2-2	12	2	Recurrence
5	37, F	LDH	RP, SD	2	9	PA, IV, PRI	5-2	22	2	None
6	48, F	Stenosis	RP	32	8	PA, PRI	2-0	2	1	None
7	44, F	Stenosis	RP	48	10	PA, IV, PRI	1-4	24	3	None
8	51, F	Stenosis	RP, SD	4	8	PA, IV	1-1	4	2	None
9	75, F	LDH	RP, FF(3), BTF(4)	5	9	PA, IV	2-1	4	1	None
10	51, M	LDH	RP	6	8	PA, IV	2-2	4	1	None

**Table 2:** Summary of the radiographic measurements of the intertransversal space at the L4/5 and L5/S1 level as well as of the optimal angle of approach at the axial level.

	Mean	Min	Max	SD
Intertransversal space L4/5^†^	21.6	16.2	25.4	2.8
Intertransversal space L5/S1^†^	10.5	3	15.4	3.9
Angle of approach^*∗*^	21°	14°	26°	21

^*∗*^Interrater reliability *r* = 0,71 indicating substantial agreement between the two raters (P. Stavrinou and C. Kabbasch).

^†^Measurements in millimeter (mm).
